# Lower Double-Wall Puncture Rate During Ultrasound-Guided Internal Jugular Vein Cannulation Using Sharper, Narrower-Gauge, and/or Length-Optimized Needles: A 6-Year Quality Improvement Clinical Series in Adult Patients

**DOI:** 10.31486/toj.22.0117

**Published:** 2023

**Authors:** James M. Riopelle, Valeriy V. Kozmenko, Melville Q. Wyche, Marion L. Yapuncich, Eddie J. Pitre

**Affiliations:** ^1^ Department of Anesthesiology, Louisiana State University Health Sciences Center–New Orleans, New Orleans, LA; ^2^ Office of Medical Education, University of South Dakota Medical Center Sanford School of Medicine, Vermillion, SD; ^3^ Department of Anesthesiology, New Orleans Veterans Administration Medical Center, New Orleans, LA; ^4^ Licensed electrician (deceased)

**Keywords:** *Cannulation*, *central venous access*, *double-wall puncture*, *jugular veins*, *needles*, *posterior wall puncture*

## Abstract

**Background:** During internal jugular vein (IJV) cannulation, needle tip injury to vulnerable subjacent cervical anatomic structures can be prevented if the cannulating needle tip is not permitted, even momentarily, to penetrate the deep portion of the IJV wall, an event known as double-wall puncture (DWP), also called posterior wall puncture.

**Methods:** We conducted a 6-year ultrasound-guided IJV cannulation quality improvement project, seeking to minimize the occurrence of DWP in 228 adult patients using needles of different gauge and tip sharpness. Most needles were length-optimized to the distance between the skin puncture site and the IJV mid-lumen for a selected angle of needle insertion by (1) using a nylon screw-on needle stop or (2) using a cannulating needle that already had the desired shaft length.

**Results:** Standard central venous cannulation kit needles were long enough to reach or traverse the deepest portion of the IJV wall in nearly all patients. Use of extra-sharp, smaller-diameter needles in place of standard needles was associated with a 26.3% relative reduction in DWP rate. Use of needles length-optimized to reach only the IJV mid-lumen was associated with a 78.4% relative reduction in DWP rate. A 0% DWP rate was attained using length-optimized 21-gauge extra-sharp needles and length-optimized 20-gauge needles of intermediate sharpness.

**Conclusion:** The 9.2% DWP rate achieved during this project was approximately half the rate reported at the time of project inception. Use of length-optimized, sharper, narrower-gauge cannulating needles may help avoid DWP during ultrasound-guided IJV cannulation.

## INTRODUCTION

Although ultrasound guidance during cannulation of the internal jugular vein (IJV) has greatly reduced the risk of inadvertent needle tip injury to the carotid artery, double-wall puncture (DWP) of this vein still occasionally results in injury to other, less-ultrasound-visible subjacent anatomic structures (Table 1).^1-16^

**Table 1.** Needle Overinsertion Injuries Reported to Have Occurred During Internal Jugular Venous Cannulation Despite Ultrasound Guidance

Injured Structure	Complications
Carotid artery^1-4^	Hematoma, stroke, arteriovenous fistula
Subclavian artery^5-7^	Hematoma, arteriovenous fistula
Vertebral artery^8-10^ (mainly pediatric)	Hematoma, pseudoaneurysm
Pleura/lung^11,12^	Pneumothorax, hemothorax
Thoracic duct^13,14^	Cutaneous chyle leak
Stellate ganglion^15,16^	Horner syndrome

Superficial anatomic landmark–guided IJV cannulation was associated with a DWP rate of approximately 50%.^17^ Adoption of ultrasound guidance reduced this rate to 17% to 20%.^18-22^ One mechanism of this reduction was the introduction in approximately 1998 of the *jab* needle insertion to help accomplish IJV single-wall puncture (SWP).^18^ Reported DWP rates, however, then remained stable at 17% to 20% for more than a decade.^23-26^

In 2012, we conducted an institutional review board (IRB)–approved IJV DWP reduction pilot study designed to determine whether SWP could be reliably accomplished by performing IJV cannulation using a (double-guidewire) microaccess technique with an extra-sharp needle. Video recordings made during that project, however, revealed that no then-available cannulating needle was sharp enough—if advanced slowly—to reliably attain SWP in adult patients.

We therefore commenced an ultrasound-guided IJV cannulation quality improvement project with the intention of achieving an IJV DWP rate lower than the prevailing rate by combining (1) use of extra-sharp narrow-gauge cannulating needles with (2) the limitation of needle tip depth during jab needle insertion through use of a mechanical needle stop.

## METHODS

Our project was conducted at a 170-bed suburban adult acute care hospital from September 2013 to January 2020, at which time the project leader was reassigned to a different facility. A total of 228 IJV cannulations were performed in operating rooms, the intensive care unit, the emergency department, or on the medical/surgical floor. All patients had a clinical indication for the procedure. Before any cannulation, the patient or a designated representative signed the hospital's standard central venous cannulation consent form. To facilitate data retrieval and analysis, we created a smart phrase that was inserted into our facility's default central venous cannulation procedure note template of the Epic electronic medical record (EMR) (Epic Systems Corporation). Alphanumeric responses to prompts for anatomic and procedural data that we deemed potentially relevant to DWP occurrence could be typed into the smart phrase (Table 2). All cannulations were performed under ultrasound guidance and using full aseptic precautions.

**Table 2.** Smart Phrase Addendum to Central Venous Cannulation Note in the Electronic Medical Record

DEMOGRAPHICS	VEIN ANATOMY	NEEDLE	OUTCOME
date:	IJ,SCL,Fem:	gauge:	Cannulation success?
pt age:	side (R/L):	tip sharpness (A-,B-,I-)	overshoot (DWP)?
pt height:	skin→superficial wall (mm):	length (mm):	# skin punctures:
pt weight:	skin→mid-lumen (mm):	insertion angle (°):	# needle re-directs:
pt sex:	skin→deep wall (mm):	tip insertion depth (mm):	notes:
operator experience:	A-P diameter (mm): M-L diameter (mm):	needle stop used? needle stop type:	

A-,B-,I-, sharpness category: A-, extra-sharp; B-, of lesser sharpness customary for vascular access; I-, of sharpness intermediate between A- and B-; A-P, anteroposterior; DWP, double-wall puncture; IJ, SCL, Fem, internal jugular, subclavian, femoral (vein being cannulated); M-L, medial-lateral; pt, patient; R/L, right/left.

At the end of the project, the IRB of the hospital where the project was implemented approved a retrospective review of IJV cannulation procedure notes to enable preparation of this report.

### Venipuncture Technique

Prior to sterile prepping and draping, we selected a skin puncture point by imaging the IJV and carotid artery in short axis around the mid neck, searching for the location with minimal overlap of the IJV over the carotid artery (parallax method).^27^ The onboard caliper function of the ultrasound device was used to measure (1) the IJV cross-sectional diameter in 2 axes (anteroposterior and medial-lateral) and (2) the depth from the skin to the most superficial point of the IJV wall (*D_superf_),* the deepest point of the IJV wall (*D_deep_*), and the IJV lumen central axis (*D_mid_*).

DWP was considered to have occurred when blood was aspirated into a syringe attached to a cannulating needle during its *withdrawal* rather than during its *advancement*.^17^

The initial phase of ultrasound-guided needle insertion was performed during short-axis imaging of the vessel during out-of-plane needle advancement. Over time, this method was increasingly supplemented with long-axis imaging of the vessel during in-plane needle advancement once the needle tip appeared to be very close to the target vein. To facilitate accurate needle aim, the traditional jab needle insertion technique for preventing DWP^18^ was modified as follows: (1) initial needle advancement was performed slowly, and (2) if no blood was withdrawn into the attached syringe when the needle tip was estimated to have reached *D_mid_*, the needle was withdrawn slightly and then re-advanced in a series of accelerating insertions until blood aspiration occurred.

If tolerated by the patient, IJV cannulation was performed in steep Trendelenburg position. In the event this maneuver failed to fully distend the target IJV, a second method of increasing intravascular pressure was added. Awake, cooperative patients were asked to perform a Valsalva maneuver with or without a voluntary 5-cm straight-leg heel lift. In mechanically ventilated patients, positive end-expiratory pressure was temporarily increased, or a sustained positive-pressure breath was administered at the moment of venipuncture.

Of the 228 cannulations, 145 were performed by the staff anesthesiologist and 83 by anesthesiology residents under his direct supervision. Two-thirds of these residents had performed fewer than 10 prior IJV cannulations. No effort was made to randomize needle selection or operator experience.

Measuring and recording aspects of IJV anatomy, using the microaccess technique, and teaching IJV cannulation technique all added to IJV cannulation time. Procedure durations, however, were not measured.

### Cannulating Needle Diameter and Sharpness

**Figure 1.**
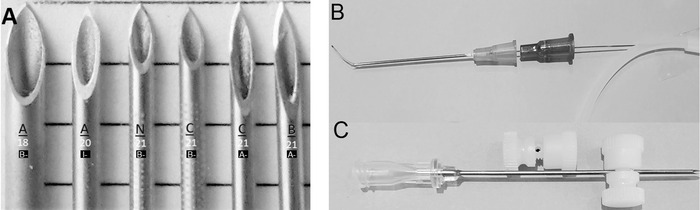
Cannulating needles used during the project. (A) Orifice views of needle tips. Black underlined letters (top) indicate manufacturer: A–Arrow-Teleflex Incorporated, N–Navilyst Medical Inc/AngioDynamics, C–Cook Medical, B–Becton, Dickinson and Company. Numbers (middle) indicate Birmingham gauge scale. White letters in black boxes (bottom) indicate sharpness categorization: A-, extra-sharp; B-, of lesser sharpness customary for vascular access; I-, of sharpness intermediate between A- and B-. Scale lines are 2 mm apart. (B) Braun 22-gauge intravenous catheter hub positioned to serve as a funnel to conduct a microaccess guidewire into the shaft of a 21-gauge Becton, Dickinson and Company needle. (C) Two nylon screw-on mechanical needle stops. The needle stop above the needle displays a hole drilled through the bolt shaft just below the bolt head through which the cannulating needle will be inserted. The lower needle stop is shown positioned along the shaft of an 18-gauge needle.

Needles used to perform IJV puncture usually had tips of greater sharpness and smaller diameter than the relatively blunt 18-gauge needles packaged in our hospital's triple-lumen central venous cannulation kit (Arrow-Teleflex Incorporated). Figure 1A shows bevel views of the tips of the needles used to perform internal jugular venipuncture during this project. Needle tip sharpness was categorized as B-bevel (B-), of lesser sharpness customary for needles used for vascular access; A-bevel (A-), extra-sharp marketed primarily for intramuscular and subcutaneous injection; and I-bevel (I-), of sharpness intermediate between B- and A-. Physical attributes of these needles are displayed in Table 3. Because 21-gauge A- needles manufactured by Becton, Dickinson and Company lack the internally tapered hub required for easy guidewire advancement into the needle shaft, internally tapered detached hubs of B. Braun Medical Inc. 22-gauge intravenous catheters were inserted into Becton, Dickinson and Company hubs prior to advancing 0.5-mm (0.018-inch) microaccess guidewires through needle shafts (Figure 1B).

**Table 3.** Attributes of Hypodermic Needles Used During Internal Jugular Vein Cannulation

	Outside Diameter		Length	Bevel Bias Angle^b^	Tip Grind Type^c^	W-t-P Silicone^d^	W-t-P Latex^d^	Sharpness	Venipunctures^f^, n=228 (122 male, 106 female)		
Manufacturer	ga^a^	mm	mm	degrees		g	g	Classification^e^	Total, n (%)	Male, n	Female, n
Becton, Dickinson and Company^g^	21	0.8	25, 38, 51	15	LP^	10.0	11.1	A-	93 (41)	45	48
Cook Medical SST-CSV^h^	21	0.8	40	14	LP^	11.0	–	A-	56 (25)	27	29
Navilyst Medical Inc/ AngioDynamics^i^ Cook Medical (SST)^i^	21	0.8	70	22	LP^	18.5	26.3	B-	37 (16)	25	12
Arrow-Teleflex Incorporated^j^	20	0.9	88	17	BB^	18.1	13.7	I-	11 (5)	7	4
Arrow-Teleflex Incorporated^k^	18	1.3	64	25	LP^	19.9	25.7	B-	31 (14)	18	13

^a^Birmingham wire gauge.

^b^Primary (first) needle grind (bias) angle, measured with respect to needle long axis using light microscopy, computerized photographic enlargement, and protractor.

^c^Needle tip geometric classifications: LP^ – lancet point with all 3 grinds on the same side of the needle tip; BB^ – back bevel point with the second and third grinds on the opposite side of the tip from the first.

^d^W-t-P is the weight in grams needed for a needle and attached syringe, perched atop an elastomeric membrane, to puncture the membrane. Membranes were 270-μm thick silicone rubber (McMaster-Carr) or 400-μm thick latex (Covidien). Each value is a mean of 5 consecutive trials performed with at least 7 needles of the same type.

^e^Nearest traditional sharpness classification, usually based on primary grind (bias) angle (A-, bias angle ∼12° [ie, intramuscular bevel]; B-, bias angle ∼18° [ie, venous bevel]), but here also classified based on our W-t-P determinations. Arrow-Teleflex Incorporated 20-gauge needles, having a nontraditional back-bevel tip grind geometry (bias angle 14°) and W-t-P values between A- and B- needles, are categorized as intermediate sharpness (I-).

^f^Number of internal jugular vein cannulations using each needle type.

^g^Marketed for intramuscular/subcutaneous injection.

^h^Extra-sharp Cook Medical Micropuncture needle.

^i^Navilyst Medical Inc/AngioDynamics and Cook Medical (SST) microaccess needles all had traditional B- tip geometry and nearly identical W-t-P measurements.

^j^Needle in the Arrow-Teleflex Incorporated central venous cannulation kit that is provided as a component of an 18-gauge catheter-over-20-gauge needle device. During this project, such needles were used without the catheter as part of the microaccess technique.

^k^Bare cannulating needle in the Arrow-Teleflex Incorporated central venous cannulation kit.

BB, back bevel point; LP, lancet point; W-t-P weight to puncture.

### Internal Jugular Vein Punctures Using Needles Functionally Shortened With Screw-On Needle Stops

For 144 of the 184 length-optimized IJV punctures, the length of the cannulating needle was optimized (shortened) to just reach *D_mid_* at the intended angle of needle insertion by attachment of a steam-autoclaved, single-use, nylon screw-on mechanical needle stop ([NS_s-o_]; W. W. Grainger, Inc, $0.70; Figure 1C).^28,29^

**Figure 2.**
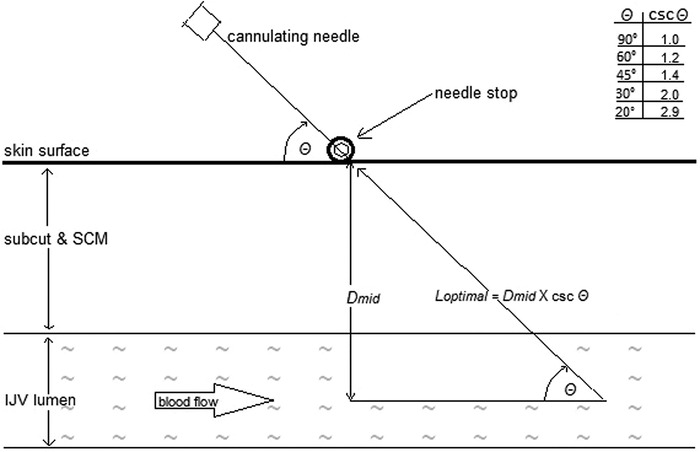
**Trigonometry of needle length optimization to limit tip depth insertion to the internal jugular vein (IJV) mid-lumen.** csc, cosecant (ratio *L_optimal_/D_mid_*); *D_mid_*, distance from the skin to the internal jugular vein mid-lumen; *L_optimal_*, optimal needle insertion distance; SCM, sternocleidomastoid muscle; subcut, subcutaneous tissue; *θ*, angle between cannulating needle and skin.

After some experience, the following formula for optimal needle shaft length (*L_optimal_*) was determined:

Equation 1 $$L_{optimal}=D_{mid}\times csc\;\theta$$

where *csc* is the cosecant (reciprocal sine) of the intended angle of needle insertion (*θ*) (Figure 2).

### Internal Jugular Vein Punctures Using Needles of Optimal Shaft Length

For 40 of the 184 length-optimized IJV punctures, needle shaft length was optimized by selecting the 21-gauge Becton, Dickinson and Company A- needle stocked by our hospital that had a shaft length closest to *L_optimal_.* Available lengths were 25, 38, and 51 mm (1, 1.5, and 2 inches). A value for *θ* was then chosen (see the table in Figure 2) that would permit the original manufacturer's hub to serve as the needle stop (NS_hub_). Eighty percent of NS_hub_ venipunctures were performed using needles with a shaft length of 25 mm.

### Frequency Distribution of Needles Used

**Figure 3.**
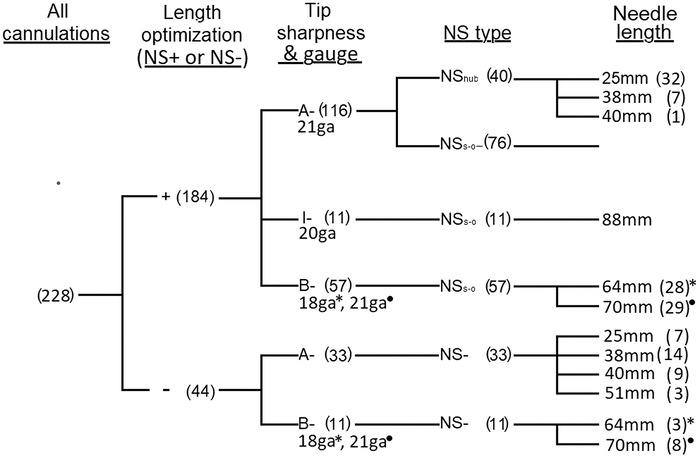
Frequency distribution of cannulating needle types used in project. Numbers in parentheses specify numbers of each type of cannulating needle used with or without a needle stop. Sharpness categories are as follows: A-, extra-sharp; B-, of lesser sharpness customary for vascular access; I-, of sharpness intermediate between A- and B-. In contrast to A- needles (always 21 gauge) and I- needles (always 20 gauge), some B- needles had 18-gauge diameter (*) and others were 21 gauge (•). ga, gauge; NS, needle stop; NS+, needle stop used; NS-, no needle stop used; NS_hub_, manufacturer's hub used as needle stop; NS_s-o_, screw-on needle stop used.

The frequency distribution of types and lengths of needles used during this project—with and without length optimization—is shown in Figure 3.

### Data Analysis

No attempted IJV cannulation was excluded from analysis. Inconsistent availability of extra-sharp cannulating needles and the means to mechanically limit their length during the 6 years of the project resulted in some cannulations being performed using standard central venous cannulation and microaccess kit needles. Of the 4,788 individual measurements that should have been recorded, 4 were inadvertently omitted: 1 patient weight and 3 IJV anatomic measurements. Substitute values for these measurements were computed using the method of mean imputation.

## RESULTS

One hundred percent of IJVs identified as patent during ultrasound imaging were successfully punctured and cannulated, but because of vessel stenosis caused by prior cannulations, advancing the tip of a cannula into the superior vena cava or innominate vein was not always possible. No IJV cannulation resulted in any clinically recognized patient harm, although on one occasion, a novice resident punctured a carotid artery with a 21-gauge needle.

Differences in the values of descriptive statistics for commonly measured demographic variables for patients in whom DWP *did* and *did not* occur were small and not statistically significant (Table 4). The *t* test *P* value for the weight difference was 0.09, and the *t* test *P* value for the age difference was 0.57.

**Table 4.** Patient Demographics by Cannulation Outcome, n=228

Cannulation Outcome	Age, years	Height, m	Weight, kg	BMI, kg/m^2^
Single-wall puncture, n=207 (91%)	62 ± 14	1.7 ± 0.1	86 ± 30	29 ± 10
Male, n=113 (55%)	62 ± 15	1.8 ± 0.1	98 ± 38	30 ± 10
Female, n=94 (45%)	62 ± 15	1.6 ± 0.1	77 ± 24	29 ± 9
Double-wall puncture, n=21 (9%)	58 ± 15	1.7 ± 0.1	81 ± 18	29 ± 9
Male, n=9 (43%)	59 ± 16	1.8 ± 0.1	86 ± 15	26 ± 4
Female, n=12 (57%)	57 ± 15	1.6 ± 0.1	77 ± 20	31 ± 12

Note: Data are presented as mean ± SD unless otherwise indicated.

BMI, body mass index.

### Anatomic Measurements Relevant to Avoidance of Double-Wall Puncture During Internal Jugular Vein Cannulation

**Figure 4.**
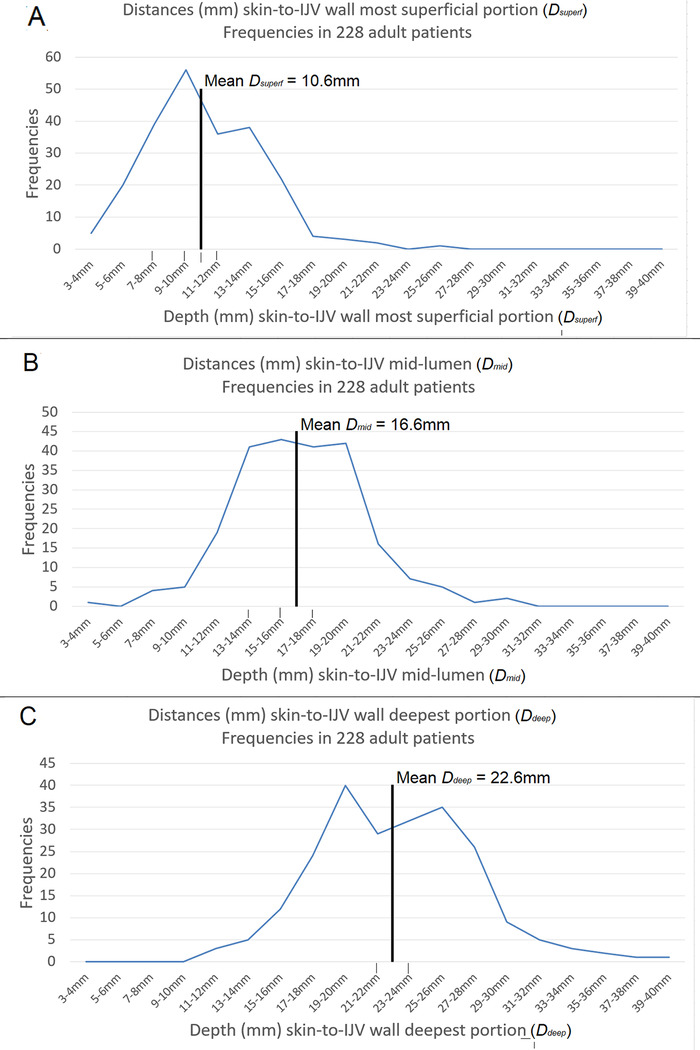
**Distribution of anatomic depth measurements for 3 points of internal jugular vein (IJV) anatomy relevant to achieving single-wall puncture during IJV cannulation.** *D_deep_*, depth from the skin to the deepest point of the IJV wall; *D_mid_*, depth from the skin to the IJV lumen central axis; *D_superf_*, depth from the skin to the most superficial point of the IJV wall.

Summary statistics for IJV anatomic measurements of the 228 adult patients are shown in Table 5. Frequency plots of the values of *D_superf_, D_mid_*, and *D_deep_* are shown in Figure 4. Equation 1 can be rearranged to determine the maximal needle tip insertion depth (*D_max__-N_*) for a needle of a specified length (*L_N_*) and at a specified angle of needle insertion (*θ*):

Equation 2 $$D_{max-N}=L_{N}÷csc\;\theta$$

**Table 5.** Summary Statistics for Measurements of Adult Patient Internal Jugular Vein Anatomy, n=228

Anatomic Variable	Mean ± SD	Minimum, Median, Maximum
Diameter		
Anteroposterior	11.6 ± 3.0	5, 11, 20
Medial-lateral	16.7 ± 4.8	6, 16, 30
Anteroposterior: medial-lateral ratio	0.7 ± 0.2	0.3, 0.7, 1.3
Distance		
Skin to superficial-most portion of wall (*D_superf_*)	10.6 ± 3.6	3, 10, 25
Skin to mid-lumen (*D_mid_*)	16.7 ± 3.9	8, 17, 30
Skin to deepest portion of wall (*D_deep_*)	22.6 ± 4.8	11, 23, 38
Mid-lumen to deepest portion of wall (*D_deep_ – D_mid_*)	5.9 ± 1.8	2, 6, 13

Note: All measurements taken perpendicular to plane of skin in mm except for (unitless) diameter ratio.

Thus, our central venous cannulation kit 18-gauge 64-mm needles—if fully inserted—were long enough to reach a depth of 45.7 mm at *θ*=45° (64 ÷ 1.4), which was >*D_deep_* in 100% (228/228) of our patients. At *θ*=30°, these needles were able to reach a depth of 32 mm (64 ÷ 2), which was >*D_deep_* in 98% (224/228) of our patients.

### Double-Wall Puncture Rates for Needles of Different Sharpness, Gauge, and/or Length

**Figure 5.**
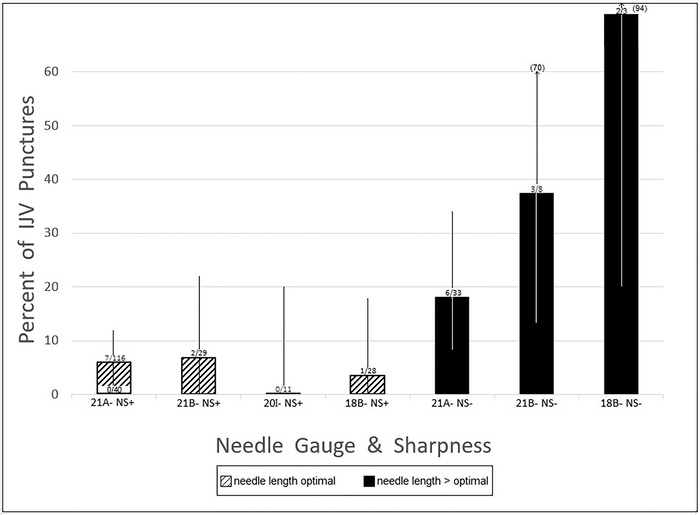
**Internal jugular vein (IJV) double-wall puncture (DWP) rates during cannulations by needle type, with and without needle stop (NS). Ninety-five percent CIs are expressed as black or white lines starting within and extending above bars. Numbers in parentheses above bars are upper limits of 95% CIs that extend beyond the range of the graph. The white bottom portion of the leftmost bar (DWP rate for 21-gauge extra-sharp NS+ needles) denotes the 0% double-wall puncture rate achieved for subcategory of 40 venipunctures performed using NS_hub_ needles (original manufacturer's hub served as the needle stop). Needle diameters are expressed using Birmingham gauge scale.** 21A-, 21-gauge, A-bevel; 21B-, 21-gauge, B-bevel; 20I-, 20-gauge, I-bevel; 18B-, 18-gauge, B-bevel; NS+, needle stop used; NS-, no needle stop used.

Figure 5 displays DWP rates and 95% CIs for each type of needle used, with or without a needle stop. Table 6 presents DWP rates for each needle type and for some needle type groups.

**Table 6.** Double-Wall Puncture Rates for Individual Needles and Some Groupings Based on Tip Sharpness, Gauge, and Length

Needle Characteristic	Double-Wall Puncture Rate, %	Double-Wall Puncture, n/n	95% CI, %
Tip Sharpness			
A-bevel, 21 gauge	8.7	13/149	4.7-14.5
I-bevel, 20 gauge	0.0	0/11	0.0-28.5
B-bevel, all gauges	11.8	8/68	5.2-21.9
21 gauge	13.5	5/37	4.5-28.8
18 gauge	9.7	3/31	2.0-25.8
Gauge			
21 gauge, any bevel, NS±	9.7	18/186	5.8-14.9
20 gauge, I-bevel, NS_s-o_	0.0	0/11	0.0-28.5
18 gauge, B-bevel, NS±	9.7	3/31	2.0-25.8
Length optimization			
NS-, all gauges	25.0	11/44	13.2-40.3
21 gauge, A-bevel and B-bevel	22.0	9/41	10.6-37.6
21 gauge, A-bevel	18.2	6/33	7.0-35.5
21 gauge, B-bevel	37.5	3/8	8.5-75.6
18 gauge B-bevel	66.7	2/3	9.4-99.2
NS+, all gauges	5.4	10/184	2.6-9.8
21 gauge, A-bevel	6.0	7/116	2.5-12.0
21 gauge, A-bevel, NS_s-o_	9.2	7/76	3.8-18.1
21 gauge, A-bevel, NS_hub_	0.0	0/40	0.0-8.8
21 gauge, B-bevel, NS_s-o_	6.9	2/29	0.9-22.8
20 gauge I-bevel, NS_s-o_	0.0	0/11	0.0-28.5
18 gauge B-bevel, NS_s-o_	3.6	1/28	0.1-18.3

Note: A-bevel, extra-sharp; B-bevel, of lesser sharpness customary for vascular access; I-bevel, of sharpness intermediate between A-bevel and B-bevel.

NS±, with or without a needle stop; NS+, with a needle stop; NS-, without a needle stop; NS_hub_, original manufacturer's hub served as the needle stop; NS_s-o_, nylon screw-on mechanical needle stop.

#### Sharpness

The DWP rate when using 21-gauge A- needles with or without a needle stop was 8.7% (13/149) vs 11.8% (8/68) when using 21- and 18-gauge B- needles with or without a needle stop. Relative risk reduction due to the use of extra-sharp needles was 26.3% ([11.8–8.7]/11.8); absolute risk reduction was 3.1% (11.8–8.7). We considered 21- and 18-gauge B- needles together based on bench test measurements indicating comparable sharpness.

#### Length Optimization

The DWP rate when using needles with some kind of needle stop was 5.4% (10/184) vs 25.0% (11/44) when using needles without a needle stop. Relative risk reduction due to length optimization was 78.4% ([25.0–5.4]/25.0); absolute risk reduction was 19.6% (25.0–5.4). The DWP rate using our central venous cannulation kit 18-gauge B- needles shortened to *L_optimal_* using NS_s-o_ was 3.6% (1/28). The DWP rate when using either 21-gauge A- NS_hub_ needles or 20-gauge I- NS_s-o_ needles was 0.0% (0/40 and 0/11, respectively). However, none of the 11 IJVs cannulated using 20-gauge I- NS_s-o_ needles had anteroposterior diameter <9 mm vs 27.5% (11/40) of IJVs cannulated using 21-gauge A- NS_hub_ needles.

### Analysis of Individual Double-Wall Puncture Events Occurring Despite Needle Shaft Length Optimization

IJV cannulation procedure note addenda written by the project leader for the 10 venipunctures associated with DWP occurrence despite needle shaft length optimization mentioned the following factors as possibly contributory to occurrence of this event (more than 1 factor was cited in several notes): resident inexperience–4; inadequately tightened NS_s-o_ slipping back upon contact with skin–2; failure of physician to arrest needle advance during jab insertion at the precise moment when the needle stop contacted skin (as evidenced by skin indentation)–5; needle aim too lateral (tip harmlessly exiting the IJV lateral wall)–1; fully distended IJV diameter ≤8 mm (third percentile)–2; severely anteroposteriorly flattened IJV that collapsed completely during inspiration in a patient with septic shock–1.

We received no complaints from colleagues or surgical suite managers related to the extra time required to make and record ultrasound anatomic measurements or perform dual guidewire microaccess IJV cannulation.

## DISCUSSION

Across the 6-year duration of this project, we achieved a DWP rate during IJV cannulation of 9.2% (21/228), approximately half the published 17% to 20% rate we had chosen as our benchmark.

### Anatomic Measurements

The principal clinical implication of the anatomic data collected during this quality improvement project is that the 64-mm (2½-inch) shaft length cannulating needles packaged in our—and other widely used—central venous cannulation kits were long enough to reach or traverse the deepest portion of the IJV wall in 98% to 100% of our patients, depending on needle insertion angle. Even the 40-mm pediatric 21-gauge cannulating needles we used would have been long enough—if fully inserted at 45°—to reach or traverse the deepest portion of the IJV wall in 90.4% (206/228) of our adult patients.

Also, the very short distance we documented in some patients between the IJV mid-lumen and the deepest portion of the IJV wall—2 mm in 1 patient; 3 mm in 15 patients—highlights the feat of manual dexterity that is sometimes required to accomplish SWP during jab venipuncture.

### Clinical Data

Because our project was not a controlled study, associations we observed between cannulating needle properties and DWP rates require confirmation.

#### Length Optimization

The needle property most strongly associated with attainment of SWP was shaft length optimization. The 3.6% DWP rate we achieved by simply using a screw-on needle stop to length-optimize the central venous cannulation kit 18-gauge 64-mm B- needles suggests that these very inexpensive devices are effective. Although we obtained a 0% DWP rate (100% SWP rate) using both 21-gauge A- NS_hub_ needles and 20-gauge I- NS_s-o_ needles, both require use of a microaccess kit, which adds $20 to $33 to the cost of central venous cannulation.

#### Length Optimization Benefit Despite DWP

Needle length optimization can possibly reduce the risk of needle tip injury to vulnerable deep cervical anatomic structures even in the event of DWP because visual and tactile feedback that occurs during needle stop skin indentation is usually sufficient to prevent needle tip overinsertion by more than a few millimeters.

#### Needle Sharpness and Gauge

The 26.3% relative reduction in DWP risk recorded using A- needles with or without a needle stop vs B- needles with or without a needle stop (13/149=8.7% vs 8/68=11.8%) and the 0% DWP rate recorded using 21-gauge A- NS_hub_ and 20-gauge I- NS_s-o_ needles together suggest that greater tip sharpness helps achieve SWP. However, because our 21-gauge A- and 20-gauge I- needles were both sharper and slimmer than the standard central venous cannulation kit needles, our clinical data cannot discern the relative importance of these 2 variables. Bench measurements suggest—at least within the diameter range of 18 to 21 gauge and the sharpness range of A- to B-—that sharpness matters more than diameter. Extra-sharp needles, however, may be more prone to dulling upon contact with bone or hard plastic.

### Other Strategies for Achieving Single-Wall Puncture

Since the start of our project, increasingly sophisticated ultrasound needle guidance methods have been reported to permit attainment of very low DWP rates using standard central venous cannulation kit needles (eg, long ± oblique vessel imaging during in-plane needle imaging).^30-32^ Such techniques, however, may require more practice than the simpler method (short-axis imaging of the vessel with out-of-plane imaging of the needle) that our residents mastered very quickly. Use of sharper, length-optimized needles would, in any case, seem to be compatible—perhaps even synergistic—with newer needle guidance techniques.

### Opportunities for Confirmation or Application of Double-Wall Puncture Avoidance Principles Described in This Report

The large differences in DWP rates recorded when performing IJV cannulations with needlessly long vs optimal-length needles may mean that a relatively small, controlled study of this variable would find a statistically significant effect. Greater confidence in the determination of SWP vs DWP could be gained through needle-in-plane ultrasound video recording during jab venipuncture.

Considering the relative infrequency of readily apparent damage to deep cervical anatomic structures resulting from needle overinsertion during IJV cannulation, demonstrating a reduction in patient injury using shorter, sharper needles would require a large clinical series. A health care system wishing to implement such a series could do so by (1) adding several anatomic and procedural variables to its EMR central venous cannulation note and (2) securing provider cooperation.

## CONCLUSION

The 9.2% DWP rate achieved during this project was approximately half the rate reported at the time of project inception. Use of length-optimized, sharper, narrower-gauge cannulating needles may help avoid DWP during ultrasound-guided IJV cannulation.

## Notes

*This article meets the Accreditation Council for Graduate Medical Education and the American Board of Medical Specialties Maintenance of Certification competencies for Patient Care, Medical Knowledge, and Practice-Based Learning and Improvement.*
